# Are Semi-Quantitative Clinical Cultures Inadequate? Comparison to Quantitative Analysis of 1053 Bacterial Isolates from 350 Wounds

**DOI:** 10.3390/diagnostics11071239

**Published:** 2021-07-12

**Authors:** Thomas E. Serena, Philip G. Bowler, Gregory S. Schultz, Anna D’souza, Monique Y. Rennie

**Affiliations:** 1 SerenaGroup Research Foundation, Cambridge, MA 02140, USA; 2 Phil Bowler Consulting Ltd., Warrington WA1 1RG, UK; philbowler.consulting@gmail.com; 3 Department of Obstetrics and Gynecology, University of Florida, Gainesville, FL 32610, USA; schultzg@ufl.edu; 4 MolecuLight Inc., Toronto, ON M5G 1T6, Canada; adsouza@moleculight.com (A.D.); mrennie@moleculight.com (M.Y.R.)

**Keywords:** semi-quantitative culture, quantitative culture, wound biopsy, chronic wounds, wound microbiology, fluorescence imaging

## Abstract

Early awareness and management of bacterial burden and biofilm is essential to wound healing. Semi-quantitative analysis of swab or biopsy samples is a relatively simple method for measuring wound microbial load. The accuracy of semi-quantitative culture analysis was compared to ‘gold standard’ quantitative culture analysis using 428 tissue biopsies from 350 chronic wounds. Semi-quantitative results, obtained by serial dilution of biopsy homogenates streaked onto culture plates divided into 4 quadrants representing occasional, light, moderate, and heavy growth, were compared to total bacterial load quantified as colony-forming units per gram (CFU/g). Light growth, typically considered an insignificant finding, averaged a clinically significant 2.5 × 10^5^ CFU/g (SE = 6.3 × 10^4^ CFU/g). Occasional growth (range: 10^2^–10^6^ CFU/g) and light growth (10^3^–10^7^ CFU/g) corresponded to quantitative values that spanned a 5-log range; moderate and heavy growth corresponded to a range of 4-log and 6-log, respectively, with a high degree of overlap in range of CFU/g per category. Since tissue biopsy and quantitative culture cannot be widely practiced and semi-quantitative analysis is unreliable, other clinically relevant approaches are required to determine wound bioburden and guide best management practices. Fluorescence imaging is a point-of-care technology that offers great potential in this field.

## 1. Introduction

Elevated levels and diversity of bacteria characterize chronic wounds, prolong healing, and increase risk of infection. The extent to which bacteria impede wound healing will vary depending on the bacterial load, species present [1], and virulence of bacteria [2,3] as well as biofilm interactions [4,5,6]. However, several lines of evidence point to 10^4^ colony forming units per gram of tissue (CFU/g) as the threshold at which healing generally begins to slow [1,7,8]; other studies show that healing is further hindered as the bacterial load increases [9,10,11]. Reducing bacterial burden through debridement, cleansing, and the application of topical antimicrobials is a basic tenet of wound care practice [12].

At present, wound care providers estimate bacterial load by a combination of clinical examination of clinical signs and symptoms and semi-quantitative cultures of wound samples. Studies have demonstrated poor sensitivity (~20%) of clinical signs and symptoms (CSS) in detecting elevated levels of bacteria [13,14,15,16,17], even for *Pseudomonas aeruginosa* [18], which is known for its hallmark symptoms. Quantitative analysis performed on tissue biopsies is considered to be the ‘gold standard’ for detecting high microbial load in chronic wounds; however, the majority of wound care practitioners are not able to take wound biopsies, and microbiological analysis is a more time consuming and costly procedure than semi-quantitative swab analysis [19,20,21]. The same is true for expensive molecular diagnostics, such as polymerase chain reaction (qPCR). Semi-quantitative microbiological analysis is lauded as being more cost effective and has gained widespread use world-wide [22]. However, the accuracy of semi-quantitative culture results compared to quantitative methods remains unclear. Few studies have made direct comparisons between the two culture methods. Some authors have suggested a linear or near linear relationship between semi-quantitative and quantitative methods [23,24]. In contrast other investigators have noted a weak correlation between the two culture methods [25,26]. The focus of this study was to compare the accuracy of semi-quantitative cultures to quantitative cultures in a large clinical trial.

## 2. Materials and Methods

Tissue biopsies were collected from 350 wounds as part of a prospective, single-blind, multicenter cross-sectional study, the fluorescence imaging and assessment guidance (FLAAG) clinical trial (clinicaltrials.gov #NCT03540004). Patients were recruited from 14 outpatient wound care centers across the United States. They were excluded if they had been treated with an investigational drug within the last month, had recently (<30 days) had a wound biopsy, had any contraindications to routine wound care, or were unable to provide consent. The trial included a minimum of 20 subjects from each major chronic wound type: diabetic foot, venous leg and pressure ulcers, and surgical wounds. The study received ethics approval by an external institutional review board (Veritas IRB, Montreal, QC, Canada).

### 2.1. Wound Biopsy, Semi-Quantitative and Quantitative Culture Analysis

Microbiological culture analysis was performed at a third-party laboratory (Eurofins Central Laboratory, Lancaster, PA, USA) that adheres to guidelines provided by the Clinical and Laboratory Standards Institute. Up to 3 biopsies were collected from the wound or peri-wound using a standard punch biopsy (6 mm in diameter). Each biopsy was then divided in half along the long axis, cut to a depth of 2 mm, and placed in sterile transportation media. Prior to analysis, 1.0 g of the biopsy was weighed out, mixed with 1 mL of Brucella broth, and homogenized for a uniform amount of time for each sample (Ultra Tissue Grinder System, Thermo Fisher Scientific, Waltham, MA, USA). Diluted biopsy homogenates were then vortexed and spread onto plates containing Blood agar/Chocolate agar (nonselective growth), Columbia CNA agar (selective gram positive), MacConkey agar (selective gram negative), or Brucella agar (for culture of anaerobes) and incubated at 35 °C in the appropriate atmosphere [15]. Note that, as these data were collected as part of a clinical trial specific to bacterial detection, only bacterial colonies proceeded to the semi-quantitative or quantitative steps; fungi were not assessed. From the 350 biopsy samples, 1053 unique bacterial isolates were identified.

**Figure 1:**
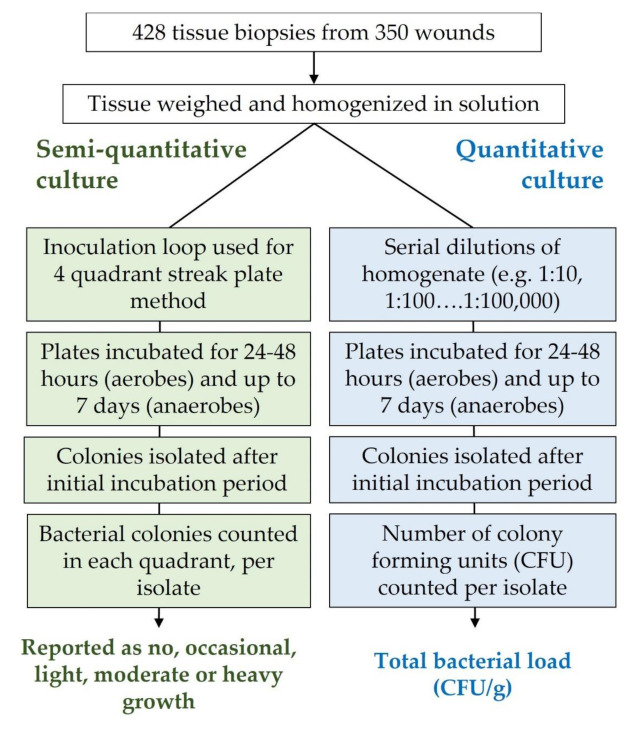
Semi-quantitative and quantitative culture methodology. In total, 428 biopsies were collected from 350 wounds; each biopsy was split in half for semi-quantitative and quantitative analysis.

Semi-quantitative analysis was performed for each isolate as summarized in Figure 1. First, aliquots of the homogenate were streaked onto several types of agar plates for pathogen identification and susceptibility. To report bacterial load, culture plates were divided into four quadrants that were then systematically streaked with the homogenate from the first to the last quadrant using an inoculation loop. After incubation for 24–48 h, the number of quadrants with bacterial growth were assessed. A result of ‘none’ was recorded if no growth was observed on the plate; ‘occasional’ was reported if growth was only observed in the first quadrant; ‘light’ was reported if growth was observed on the first and second quadrants; and ‘moderate’ was reported if growth was observed in the first, second, and third quadrants. A result of ‘heavy’ was reported if growth was observed on all four quadrants of the agar plate.

**Figure 2:**
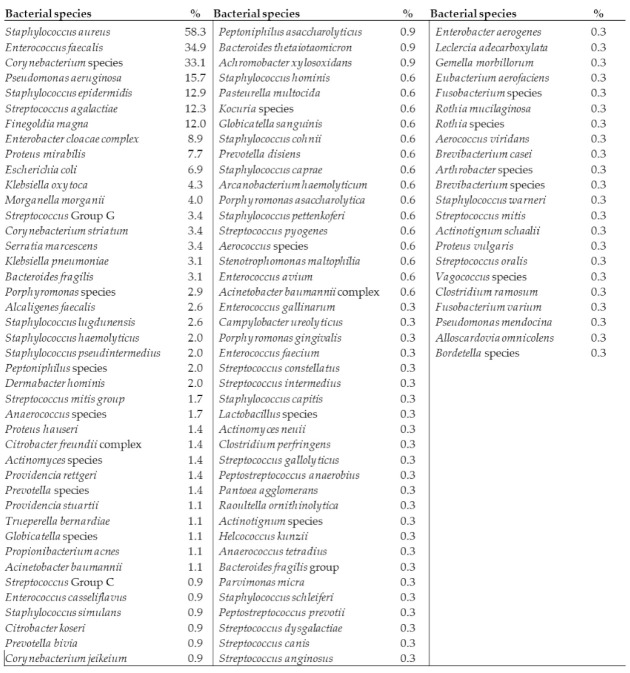
Prevalence of bacterial species detected. Percent denotes frequency of each species detected across the 350 wounds analyzed.

For quantitative analysis (Figure 1), the homogenate solution was serially diluted in saline solution (1:10 to 1:10,000), transferred onto agar plates, and incubated overnight and then monitored every 24 h for up to 7 days. After incubation, colonies were counted and recorded from each plate that had grown between 0–100 colonies. Colony forming units per gram (CFU/g) were calculated by multiplying the recorded value by the dilution factor then multiplying by the correction factor for the weight of the biopsy and the partial volume of homogenate solution that was plated. Distribution of the CFU/g bacterial loads corresponding to the semi-quantitative categories was statistically analyzed by log transforming the data, performing linear regression, and the Mann–Whitney and Wilcoxon tests; outlier rejection was performed resulting in the removal of 112 out of 1053 data points. To identify bacterial species (Figure 2), matrix assisted laser desorption ionization-time of flight mass spectrometry (MALDI-TOF; Bruker Daltonics, Billerica, MA, USA) was used [27].

### 2.2. Fluorescence Imaging Procedure

As part of the FLAAG trial, clinicians performed fluorescence imaging for point-of-care detection for elevated bacterial burden. The non-contact imaging procedure captures and displays fluorescence signals from bacteria and tissue on a handheld device to identify any regions in and around the wound with elevated bacterial burden. The imaging device (MolecuLight i:X, Toronto, ON, Canada) visualizes fluorescence from bacteria in and around the wound using a safe violet (405 nm wavelength) light. Red fluorescence is detected from at least 28 porphyrin-producing bacterial species (e.g., *Staphylococcus* spp., *Enterobacter* spp., *Proteus* spp.) including Gram positives, Gram negatives, aerobes, and anaerobes at loads of >10^4^ CFU/g [28], while cyan fluorescence is associated with the presence of *Pseudomonas aeruginosa* [18]. Tissue appears green due to matrix components [29].

## 3. Results

A total of 428 biopsies were collected from 350 patients (125 females, 225 males) at 14 outpatient wound care centers across the United States. Wound types included diabetic foot ulcers (138), pressure ulcers (22), surgical sites (60), venous leg ulcers (106), or other various etiologies (24). From the 428 biopsies collected, 1053 bacterial isolates were cultured. In total, 106 bacterial species were identified, 78 of which were aerobes (73.5%) and 28 were anaerobes (26.4%); 68 were Gram positive and 38 were Gram negative. Staphylococcus aureus was the most prevalent bacterial species detected, present in 58% of the study wounds (Figure 2). Fungal analysis was not a goal of this study, however there were two yeast species detected.

**Figure 3:**
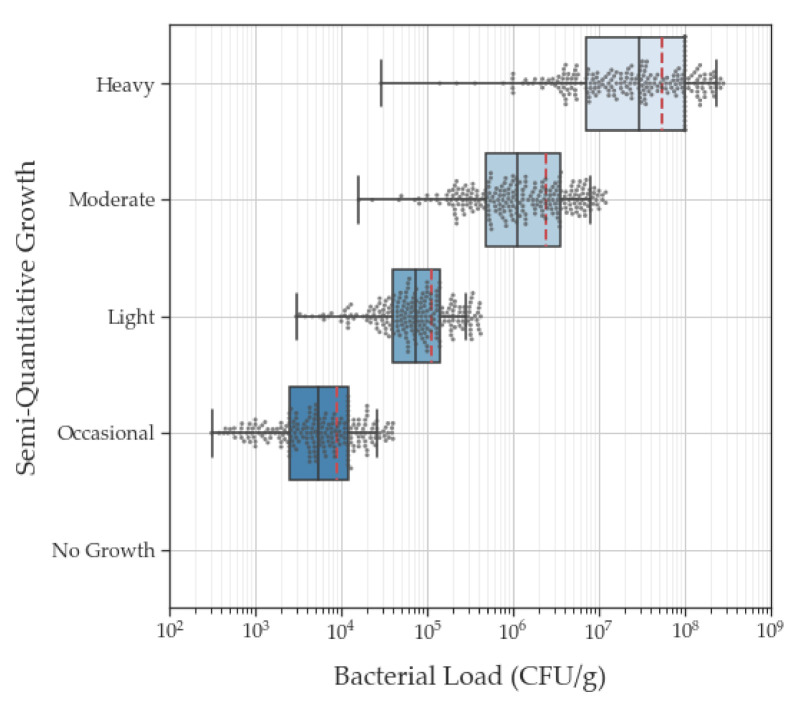
Box and whisker plot of semi-quantitative and quantitative culture results for each bacterial isolate (*n* = 941 after outlier rejection). The box lines indicate 25th, 50th (i.e., median, bold text), and 75th percentiles. Quantitative values represent log transformed bacterial loads. Error bars indicate minimum and maximum values. Red dashed lines indicate mean value. Grey dots represent individual data point. Grey vertical lines and black ticks on *x*-axis denote subdivision units between each major log10 division.

Linear regression analysis comparing the results of the semi-quantitative and quantitative culture analysis of all isolates after the log transformation of the quantitative data and outlier removal demonstrated a statistical correlation (r = 0.85) between these culture methods (Figure 3). Despite the clear correlation between these analysis methods, we found a wide range of bacterial loads in each semi-quantitative category with considerable overlap between categories (Table 1). Occasional growth (range: 10^2^–10^6^ CFU/g) and light growth (10^3^–10^7^ CFU/g) corresponded to quantitative values that spanned a 5-log range. Moderate growth (10^4^–10^6^ CFU/g) corresponded to quantitative values that spanned a 4-log range. Heavy growth (10^4^–10^8^ CFU/g) corresponded to bacterial loads spanning a 6-log range.

It is important to note that 93.5% of the isolates categorized as having ‘light growth’ had quantitative bacterial loads of 10^4^−10^5^ CFU/g (Table 2); these are loads that have repeatedly been associated with delayed wound healing [7,8]. Of the 1053 isolates, 40 isolates (3.7%) yielded no growth and had bacterial loads of 0.0 CFU/g. Almost half (44.3%) of the isolates within ‘light growth’ (*n* = 246) had quantitative bacterial loads of >10^5^ CFU/g, and the average (standard error) quantitative bacterial load of isolates within the ‘light growth’ category was 2.5 × 10^5^ CFU/g (6.3 × 10^4^ CFU/g, Table 1). A significant proportion (37.0%) of isolates categorized as ‘occasional growth’ (*n* = 238) had quantitative bacterial loads of 10^4^ CFU/g. There were 117 isolates with quantitative bacterial loads between 10^5^–10^9^ CFU/g that were categorized as having only ‘occasional growth’ or ‘light growth’ based on semi-quantitative culture results. Quantitative bacterial loads of 10^5^–10^6^ CFU/g had significant overlap in semi-quantitative categories; in some instances (10.3% of total isolates), these loads corresponded to ‘light’ growth, while in others, they corresponded to moderate (21.6%) or heavy growth (6.7%). Altogether, these findings demonstrate that semi-quantitative cultures provide poor consistency or certainty regarding which wounds harbor bacterial loads that warrant intervention.

**Figure 4:**
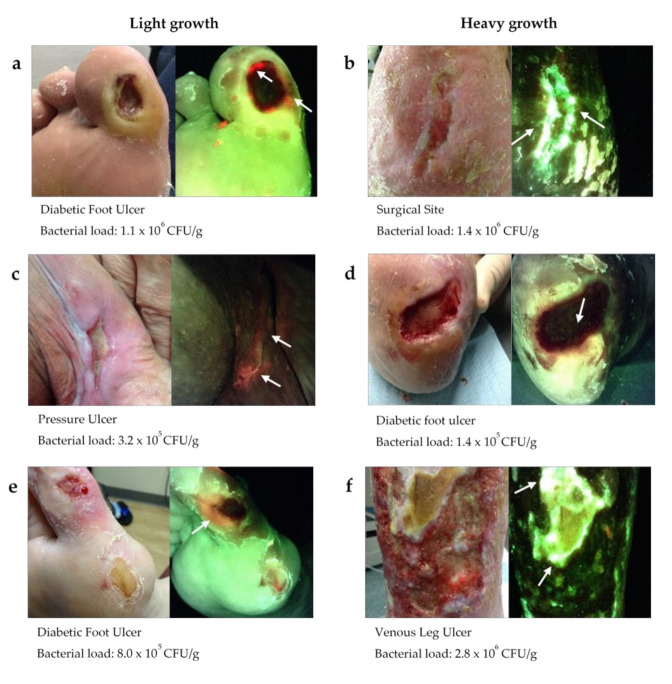
Wounds with bacterial loads of 10^5^–10^6^ CFU/g via quantitative culture where ‘light’ or ‘heavy’ growth was indicated via semi-quantitative culture. Maximum bacterial load obtained by quantitative culture analysis is listed below each example. Fluorescence images (MolecuLight i:X) revealed red or cyan fluorescence (denoted with white arrows) indicative of bacterial loads >10^4^ CFU/g in all wounds listed above. Regions of cyan in panels (**b**,**f**) were confirmed as *Pseudomonas aeruginosa* in these cases. Green is indicative of matrix components from tissue [29].

The disparity in results between semi-quantitative and quantitative cultures is further illustrated in Figure 4. Quantitative cultures from each of these wounds revealed total bacterial loads of 10^5^ to 10^6^ CFU/g; however, the wounds in the left panel were all categorized by semi-quantitative cultures as ‘light growth’ while the wounds on the right panel were categorized as ‘heavy’ growth. For each of these wounds, fluorescence images of bacterial burden (MolecuLight i:X, Toronto, ON, Canada) captured immediately prior to biopsy revealed the presence of red or cyan fluorescence signals, indicating bacterial loads of >10^4^ CFU/g [15,30].

## 4. Discussion

The determination of elevated microbial load and infection status in chronic wounds has challenged wound specialists throughout the modern wound care era. This study, the largest comparative analysis of semi-quantitative and quantitative culture methods to date, shows that semi-quantitative culture is an inconsistent and therefore unreliable method of determining bacterial load in wounds. For all semi-quantitative categories, bacterial loads spanned 3–5 logs. In the range of ‘light growth’ (10^3^ to 10^7^ CFU/g) bacterial loads spanned from ‘no concern’ (<10^4^ CFU/g; <10% of the time) to increased risk of delayed healing and graft failure (10^4^ CFU/g; 51.6%) and to levels suggestive of invasive infection requiring intervention (>10^5^ CFU/g; 44.3%). In addition, 93.5% of ‘light growth’ wound microbiology reports correspond to levels that have been associated with impaired healing (10^4^ and 10^5^) [7,8].

These findings are consistent with those of Gardener et al. who evaluated the accuracy of semi-quantitative swab cultures [31] and highlighted the limitations and imprecision of semi-quantitative culture compared to quantitative methods. The results herein demonstrate that semi-quantitative cultures produce a highly variable range of bacterial loads within each category.

A limitation of this study was that it focused on semi-quantitative and quantitative analysis alone and did not consider the impact of bacterial species present in terms of virulence expression and pathogenicity. For example, a low bacterial load of *Pseudomonas aeruginosa* may have been more detrimental to wound healing than a moderate to heavy load of *Escherichia coli*. Additionally, the methods used for semi-quantitative and quantitative analysis may have missed fastidious species that are difficult to culture, and as such, these species may have been underrepresented in the current analysis [32].

In the clinical setting, topical antiseptics and systemic antibiotics are often prescribed based on initial clinical signs and symptoms. However, there is often uncertainty regarding the presence of these symptoms, leading clinicians to rely on microbiological analysis, which is considered to be more definitive. Treatment pathways will then be determined based on the combined clinical and microbiological information available. Reliance on highly variable and unreliable semi-quantitative culture data to support best practice may contribute to the inappropriate use of antibiotics. As demonstrated in Figure 4, despite having approximately equivalent bacterial loads, some wounds were categorized as ‘heavy’ growth and others ‘light’ growth. A culture report indicating ‘heavy’ growth is considered to have a higher risk profile for infection than light growth, and thus may be more likely to receive antibiotics. Alternatively, a result of heavy growth that may in fact reflect low quantitative bacterial loads may lead to an unnecessary prescription of antibiotics. Inaccurate diagnosis of bacterial burden may lead to treatment being withheld or applied in error [33]. It is well known that overuse of antibiotics is a key driver of antibiotic resistance [34,35,36]. This is of particular concern among elderly patients due to the increased rate of antibiotic prescription [37] and the risk of adverse effects of antibiotic treatment in this population [38]. Prior to prescribing antibiotics, bacterial removal should be attempted through more traditional strategies (e.g., cleansing, debridement, and topical antimicrobial agents). Though there is agreement that any complex, non-healing wound with devitalized tissue is likely to harbor significant and multi-species bioburden warranting treatment, management approaches vary widely. Often, systemic antibiotics are prescribed even when infection status is ambiguous. Alternatively, if the microbiological results are inconsistent, the clinician may opt to run additional tests before prescribing antibiotics, further delaying appropriate treatment. Information regarding wound bioburden is extremely important to the wound care practitioner to apply appropriate wound hygiene and to remove as much of the bioburden as possible.

The Joint Commission, which evaluates and accredits U.S. health care organizations, has mandated an antimicrobial stewardship plan (ASP) in all outpatient wound clinics in the US. Unfortunately, wound specialists in most clinics lack the tools to make an accurate diagnosis of clinically significant bacteria in chronic wounds. Here we show that semi-quantitative cultures are unreliable, as they lack the sensitivity to guide antimicrobial therapy. Occasional or light growth on semi-quantitative culture does not mean the wound is free of elevated bacterial levels. While a quantitative culture of biopsies is more accurate than a semi-quantitative culture, it is rarely performed, as it is more expensive, time consuming, and requires expertise in biopsy collection. In addition, bacteria within a biofilm can develop a culture-resistant phenotype, making the detection and identification of bacteria within biofilms a challenge [39,40]. Molecular techniques (i.e., qPCR, DNA pyrosequencing) can address these limitations and provide a more accurate report of the diversity of the microbial population [41,42,43] but are less practical to implement due to complexity, costs, and availability. Alternative methods for the detection of bacterial burden at point-of-care are needed.

### Proposed Solution

Among the emerging accessible point-of-care diagnostic solutions for the detection of bacterial burden and/or biofilm are bacterial protease activity [44], biofilm blotting [45], and the real-time fluorescence imaging of elevated bacterial burden (Figure 4) [15,30]. Detection of a serine protease produced by *Staphylococcus aureus* has been used to determine its presence in chronic wounds and has recently been shown to predict a wound’s probability of healing [44]. However, this type of analysis does not provide an indication of infection status, nor does it provide information on the location of the bioburden to guide wound hygiene and best practice. Biofilm blotting provides locational information, but the evidence for its impact on treatment plan and outcomes based on this early-stage technology is limited [36]. In contrast, fluorescence imaging can accurately determine the location and extent of wound bioburden at the point-of-care. The FLAAG clinical trial, from which data from this study was derived, demonstrated that point-of-care fluorescence imaging increases detection of significant levels of bacteria (>10^4^ CFU/g) in wounds by 4-fold compared to clinical signs and symptoms [15]. Additional studies also demonstrate the ability for this point-of-care device to detect bacteria within wound biofilms [46]. It could be argued that the increased detection of bacterial bioburden and biofilm might prompt the inappropriate use of antibiotics at the detriment of antimicrobial stewardship efforts. However, evidence in several recent studies has shown that routine fluorescence imaging leads to more thorough and effective wound hygiene (i.e., cleansing and debridement) [15,47,48,49,50]. Information on the presence and location of the bioburden is key to wound hygiene efforts, but the particular species present need not be known for its removal. Encouragingly, fluorescence image-informed wound hygiene efforts have been shown to reduce use of systemic antibiotics and topical antiseptics while concomitantly increasing wound healing rates [50]. This technology has therefore been suggested as a solution to meeting the Joint Commission requirements for an antimicrobial stewardship program [51].

## 5. Conclusions

Clinicians agree that intervention is needed for non-healing, complex wounds, but the extent of treatment may vary widely. At present, treatment selection is reliant on clinical expertise and, if needed and available, diagnostic information obtained from wound sampling and/or other methods. Semi-quantitative microbiological analysis of wound swab samples is widely used and is relatively inexpensive compared to biopsy samples and fully quantitative microbiological analysis methods. However, the high degree of variability of semi-quantitative analysis shown in this study from ‘gold standard’ biopsies suggests that this microbiological technique may have diminished value in guiding clinical decision making in chronic wounds. Alternative diagnostic methods described above may provide a solution to the diagnosis of clinically significant bacteria in chronic wounds.

Clinical and microbiological information should be used to guide the treatment of wounds. Addressing the problem of biofilm in hard-to-heal wounds should also be considered. Early recognition and definitive treatment of wound bioburden is essential in managing the misuse of antimicrobials in the field of wound care.

## Figures and Tables

**Table 1:** Mean (standard error) and median (minimum, maximum) quantitative culture values corresponding to occasional, light, moderate, or heavy categories of semi-quantitative culture. Values reflect all data points (*n* = 1053; no outlier rejection).

Semi-Quantitative Culture Categories	Quantitative Culture Values	
	**Mean (SE)**	**Median (Range)**
Occasional	4.9 × 10^4^ CFU/g (3.1 × 10^4^ CFU/g)	6.6 × 10^3^ CFU/g (3.1 × 10^2^ CFU/g–7.3 × 10^6^ CFU/g)
Light	2.5 × 10^5^ CFU/g (6.3 × 10^4^ CFU/g)	8.6 × 10^4^ CFU/g (3.0 × 10^3^–1.4 × 10^7^ CFU/g)
Moderate	5.4 × 10^6^ CFU/g (6.1 × 10^5^ CFU/g)	1.6 × 10^6^ CFU/g (1.6 × 10^4^–7.3 × 10^7^ CFU/g)
Heavy	1.4 × 10^8^ CFU/g (2.9 × 10^7^ CFU/g)	3.5 × 10^7^ CFU/g (2.9 × 10^4^–7.0 × 10^9^ CFU/g)

**Table 2:** Distribution of bacterial loads per semi-quantitative culture category. Values represent the number of isolates within each semi-quantitative category with the corresponding bacterial load reported by quantitative culture. N represents the total number of isolates per semi-quantitative category. 40 isolates yielded ‘no growth’ and bacterial loads of 0 CFU/g.

	Semi-Quantitative Category			
Quantitative Bacterial Load	Occasional N = 238	Light N = 246	Moderate N = 272	Heavy N = 257
<10^4^ CFU/g	142	10	0	0
10^4^ CFU/g	88	127	8	1
10^5^ CFU/g	7	103	99	7
10^6^ CFU/g	1	5	128	64
10^7^ CFU/g	0	1	37	97
>10^8^ CFU/g	0	0	0	88

## Data Availability

Not applicable.
